# Editorial: Macrophages Role in Integrating Tissue Signals and Biological Processes in Chronic Inflammation and Fibrosis

**DOI:** 10.3389/fimmu.2017.00845

**Published:** 2017-07-21

**Authors:** Tarcio Teodoro Braga, Ivan C. Moura, Ana Paula Lepique, Niels Olsen Saraiva Camara

**Affiliations:** ^1^Immunology Department, University of São Paulo, São Paulo, Brazil; ^2^Institut national de la santé et de la recherche médicale (INSERM), Paris, France; ^3^Nephrology Division, Medicine Department, Federal University of São Paulo, São Paulo, Brazil; ^4^Renal Physiology Laboratory, Faculty of Medicine, University of São Paulo, São Paulo, Brazil

**Keywords:** chronic inflammation, fibrosis, macrophage plasticity, macrophage subtypes, homeostasis maintenance

**Editorial on the Research Topic**

**Macrophages Role in Integrating Tissue Signals and Biological Processes in Chronic Inflammation and Fibrosis**

Macrophages comprehend a population with wide range phenotypes and roles in homeostasis maintenance and diseases. Technology improvements enable researchers to track different macrophage populations in different tissues and situations and hypothesize on their role in promoting inflammation or maintaining tissue homeostasis. In the present editorial, we present a concise series of discussions on the role of these cells, its response to the microenvironment, and effects on other cells during tissue injury and repair. We also discuss the themes proposed by the authors on macrophage plasticity during fibrotic processes in the context of the topic subject. M1 macrophages are considered foe cells for the pro-fibrotic process once they are associated with pro-inflammatory functions (Braga et al.), and an exacerbation of tissue inflammation initiates the pro-fibrotic process (1). On the other hand, M2 macrophages have anti-inflammatory properties due to its ability to secrete IL-10, arginase, and TGF-β (2). However, when the insult is persistent, excessive M2 macrophage activation leads to continuous TGF-β production, promoting increased extracellular matrix deposition (3). In this scenario, despite its friendly behavior against the exacerbated fibrosis development, M2 becomes foe cells in the tissue repairing. Macrophages are also able to influence innate lymphoid cells (ILCs) during the fibrotic process (Hams et al.). Repetitive cycles of epithelial damage and repair are able to generate fibrosis through the release DAMPs and alarmins by epithelium (4). Among the alarmins, IL25, IL33, and TSLP are able to polarize ILCs to the ILC2 phenotype. ILC2 can enhance Th2 responses and collagen deposition (5, 6), either indirectly *via* IL13-mediated dendritic cell priming or directly through CD4-T cells interaction (*via* MHCII-CD4) (7, 8). In addition, ILC2 produces IL4 and IL5 and induces tissue collagen deposition in pulmonary and hepatic models of fibrotic diseases (9, 10). In turn, deficiency of IL25 and IL33 or their receptors, IL17RB and ST2, respectively, leads to decreased collagen deposition (5, 9). However, the apparent redundancy of these alarmins may be due to different ligand and receptor expression at different anatomical sites (11).

ILC2s interact with macrophages on the improvement of obesity-induced insulin resistance (Castoldi et al.). Different subtypes of macrophages are related to the maintenance of adipose tissue (AT) homeostasis during the lean state, obesity, and insulin resistance (Castoldi et al.). It has been known that the microenvironment in a lean AT is composed of macrophages subtypes in a ratio of 4:1 M2:M1 (12). To maintain AT homeostasis in this lipid-rich microenvironment, macrophages present increased adiposity (13) and increased expression of fatty acids transporters (13). However, obesity status triggers the accumulation of M1 macrophages, although it was reported that the secretion of pro-inflammatory cytokines in AT is dependent on peroxisome proliferator-activated receptor gamma (PPAR-γ), an M2 marker (14). Inflammatory factors present in obesity context lead to insulin resistance, characterized by decreased phosphorylation of insulin receptor substrate-1 and -2, decreased phosphorylation of Akt (15, 16) and activation of the mammalian target of rapamycin signaling pathway (17), a sensor of nutrients able to alter the cellular metabolism. In obesity, nutrient sensing by mTOR regulates the switch of ATMs from M2 to M1 (18). However, obesity can be controlled through the production of large amounts of anti-inflammatory cytokines and the induction of uncoupling protein 1 expression in AT, a process called “beiging” or “browning” (19). In line with the relationship between AT and inflammation, it has been reported high levels of inflammatory mediators in the context of cachexia (de Matos-Neto et al.), a health problem present especially in cancer patients (20). Weight loss, the most visible feature of cachexia, is accompanied by increased production of CCL2, CCL3, TNFα, and IL1β and reduced relative numbers of M2 macrophages in the tumor environment (de Matos-Neto et al.).

Macrophages directly influence the metabolic status of the organism (21). Different sterile inflammation, in special type 1 diabetes (T1D) can be triggered by leukotriene B4 (LTB4) (Filgueiras et al.). Filgueiras et al. wonder if LTB4 could be targeted in new therapy strategies for treating T1D once LTB4 could either increase pro-IL1β expression or potentiate the IL1R activation by modulating MYD88. Previously, the same group has demonstrated that low insulin concentrations are able to induce LTB4 production, which triggers systemic inflammation through MyD88 and its transcriptional effector STAT-1 (signal transducer and activator of transcription 1) (22). On the other hand, insulin-treated mice showed less LTB4 in the blood and reduced Myd88 and Stat1 expression in macrophages. In addition, diabetic mice lacking 5-lipoxygenase or the receptor for LTB4 produced less pro-inflammatory cytokines (22). Mitochondrial DNA (mDNA) derived from diabetic mice is also implicated in the activation of NLRP3 and IL1β in the context of T1D (Carlos et al.). It has been known that NLRP3 deficiency plays a protective role against T1D (23) and that polymorphisms in NLRP3 are associated with T1D (24), however, the precise mechanisms by which NLRP3 is triggered in the context of T1D was poorly explored. Besides demonstrating the importance of NLRP3 for the development of T1D, Carlos et al. also took advantage of a sub dosage model of disease that is not able to induce T1D, unless mDNA was given concomitantly with streptozotocin. However, it is still puzzling the fact that only mDNA from diabetic mice activates the NLRP3 inflammasome.

Besides homeostasis-altering compounds, exogenous molecules can also alter the macrophage status of activation (25). Crystalline silica reduces the activation of macrophages by reducing TLR2 expression (Beamer et al.). Previous studies established that the scavenger receptor CD204 is important for the binding/uptake of silica (26, 27). It has been also demonstrated that silica crystals activate NLRP3 inflammasome and induce IL1β production (28), a mechanism dependent of the first signal triggered by the TLR4 agonist, LPS. Beamer et al. demonstrated, on the other hand, that silica crystals leads to less IL1β production after Pam3CSK4 and Pam2CSK4 stimulus, lipopeptides recognized by the TLR2/1 and TLR2/6 heterodimer, respectively (Beamer et al.). Tissue-resident intestinal macrophages can also contribute to the gut homeostasis by eliminating invading pathogens without inducing a robust inflammatory response (Kühl et al.). Bone marrow-derived monocytes are the precursor cells of tissue-resident intestinal macrophages (29) and in the context of ulcerative colitis (UC) and Crohn’s diseases (CD), increased numbers of M1 macrophages are observed despite monocyte infiltration. In addition, lesions of UC, but not CD, are characterized by impaired bacterial clearance, formation of granulomas, inflamed mesenteric fat tissue, and pronounced fibrosis.

The prevention of damage that would be caused by macrophage prolonged activation is achieved by changes in their transcriptional program (Hamidzadeh and Mosser). ATP and adenosine can diminish the production of inflammatory cytokines by macrophages (30). In an inflammatory scenario, TLR-stimulated macrophages undergo metabolic alterations that result in an increase rate of aerobic glycolysis and production of ATP. This nucleotide is rapidly hydrolyzed to adenosine on the macrophage surface by CD39 and CD73 (30). Following TLR stimulation, macrophages dramatically upregulate their expression of receptors for adenosine, in a physiological self-regulating program. In addition, it has been demonstrated that IFNγ sustains macrophage inflammatory responses, by attenuating their sensitivity to extracellular adenosine (31). This decreased macrophage sensitivity to adenosine delays the transition of macrophages to a regulatory phenotype, allowing them to sustain macrophage activation for the duration of an adaptive immune response. IFNγ-mediated adenosine sensitivity signals through STAT1 (31); however, the exact mechanism whereby IFNγ affects the macrophage activation remain to be enlightened. However, when not controlled, blood-borne infections change the splenic microenvironment and can ultimately lead to splenomegaly (32). Splenic architecture and differences among red pulp (RpMΦs), marginal metallophilic (MMMΦs), and marginal zone macrophages (MZMΦs) were described by Borges da Silva et al. CD47, a self-molecule ubiquitously expressed on many cell types, function as an inhibitory signal for phagocytosis (33) and red blood cells expressing a modified isoform of CD47 are phagocytized by RpMΦs (34). MZMΦs and MMMΦs populate the interface between the bloodstream and lymphocyte-rich zones, and for this reason they are candidate cells to bridge innate and adaptive immunity. In this collection of articles, the authors show how macrophages influence chronic inflammatory diseases, and how the understanding of their biology can contribute to improved scenario for balance the homeostasis. We hope this collection can help further studies on the development of new therapies and in the better understanding of the biology of these cells.

## Author Contributions

TB wrote the manuscript. IM, AL, and NC helped to evaluate and edit the manuscript.

## Conflict of Interest Statement

The authors declare that the research was conducted in the absence of any commercial or financial relationships that could be construed as a potential conflict of interest.
